# Supplementary material for: Coordination of transporter, cargo, and membrane properties during non-vesicular lipid transport
Source: Commun Biol. 2024 Nov 27;7:1585. doi: 10.1038/s42003-024-07301-3 (PMC11603022; doi:10.1038/s42003-024-07301-3)
Supplement: Supplementary file 2 — Supplementary Information [file 42003_2024_7301_MOESM2_ESM.pdf]

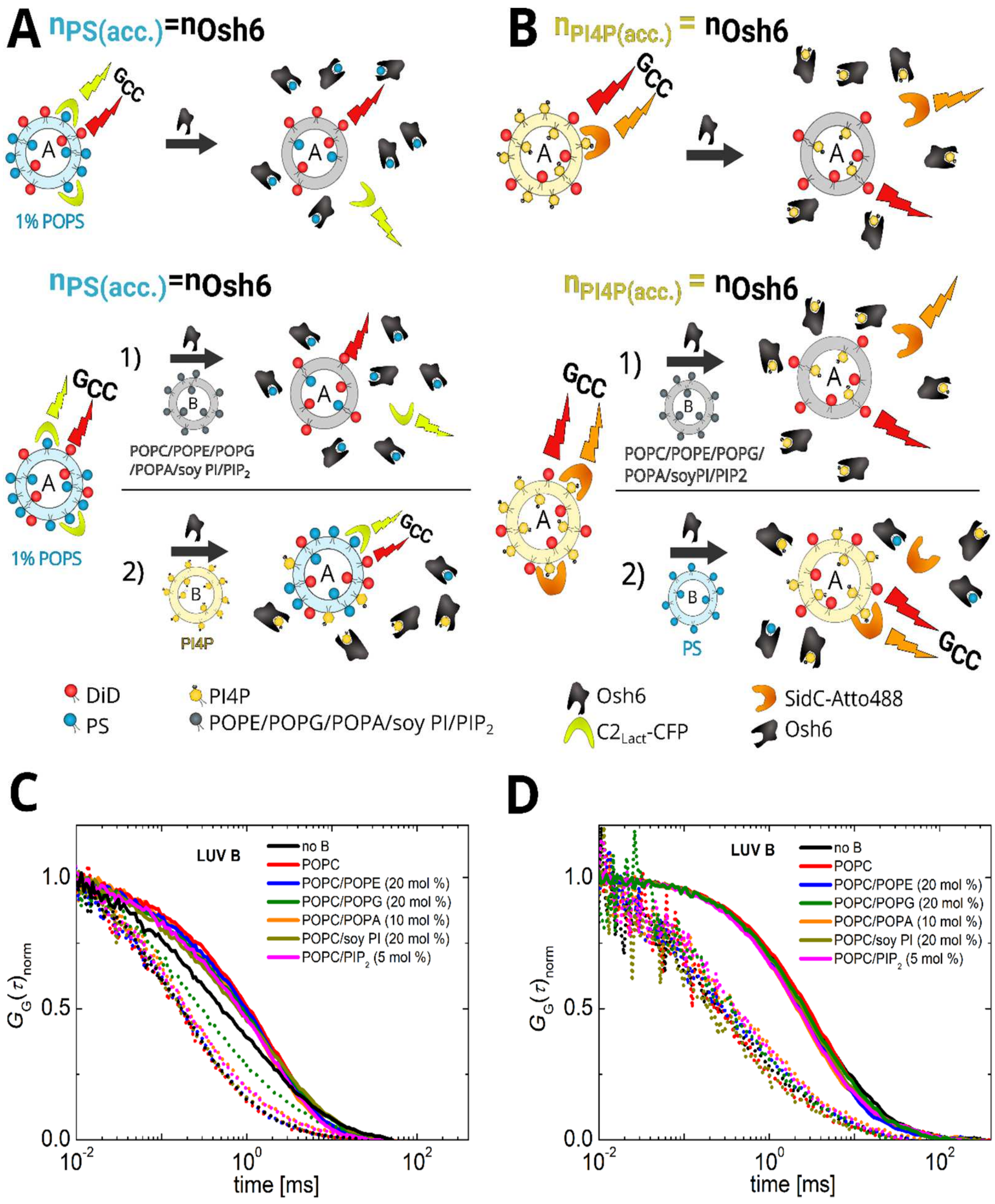

Figure S1. Extraction versus transport. Inspection of the status of the biosensor before and after the extraction experiment. A) Scheme of the PS extraction assay. Upper: The amount of accessible PS (n_{PS}(acc.)) equals the amount of Osh6. The FCCS read-out parameter ($G_{cc}(0)/G_R(0)$, simply G_{cc}) monitoring the mutual motion of DiD and C2$_{Lact}$ fused to CFP drops upon Osh6 addition. Lower: Scheme of the assay when the PS donating LUVs are in presence of other, PS-free LUVs either not bearing a competitive ligand (1) – PS extraction occurs or bearing a competitive ligand (2) – PS extraction is compromised. B) Scheme of the PI4P extraction assay. Upper: The amount of accessible PI4P (n_{PI4P}(acc.)) equals the amount of Osh6. The FCCS read-out, G_{cc}, monitoring the mutual motion of DiD and SidC-Atto488 disappears upon the Osh6 addition. Lower: Scheme of the assay when other, PI4P-free LUVs (marked as B) are present during the extraction. The LUVs B either do not contain the competing cargo (1) – the extraction is not affected or contain the competing cargo (2) – the

extraction is compromised. C) Comparison of the normalized autocorrelation curves of $C2_{Lact}$-CFP before (solid lines) and after (dotted lines) the extraction for various LUVs B added to the extraction mixture: no LUVs B (black), LUVs B composed of POPC (red), POPC/POPE (20 mol %) (blue), POPC/POPG (20 mol %) (green), POPC/POPA (10 mol %) (orange), POPC/soy PI (20 mol %) (dark yellow), POPC/PIP_2 (5 mol %) (magenta). D) Comparison of the normalized autocorrelation curves of SidC-Atto488 before (solid lines) and after (dotted lines) the extraction for various LUVs B added to the extraction mixture: no LUVs B (black), LUVs B composed of POPC (red), POPC/POPE (20 mol %) (blue), POPC/POPG (20 mol %) (green), POPC/POPA (10 mol %) (orange), POPC/soy PI (20 mol %) (dark yellow), POPC/PIP_2 (5 mol %) (magenta).

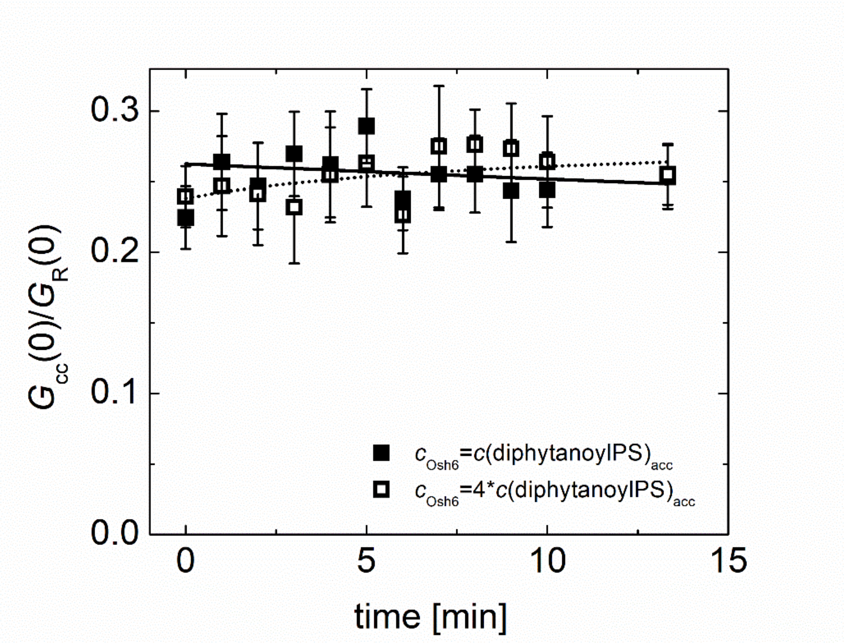

Figure S2. Extraction of non-extractable lipid – diphytanoylPS (phPS) by Osh6. Temporal drop in G_{cc} during the phPS extraction. The extraction from LUVs containing 1 mol % phPS was carried out by equimolar concentration of Osh6 (full squares), and by fourfold excess of Osh6 (hollow squares). The initial values of $G_{cc}(0)/G_R(0)$ here (~0.25) are larger than those shown in Fig. 2D (~0.08). This discrepancy may arise from several factors: i) the sensitivity of the biosensor $C2_{Lact}$-CFP towards phPS may differ due to the lipid headgroup being variably buried in the membrane; ii) the amount of DiD incorporated in the tested LUVs may vary since the lipid area of phPS differs from that of PS; and iii) LUVs, as a self-assembly system, can slightly vary between individual preparations, resulting in differences in the total number of LUVs and the amount of incorporated lipids from one preparation to another. All of these factors impact the absolute value of the G_{cc} read-out. Therefore, rather than focusing on absolute numbers, we follow the temporal evolution of G_{cc}. *All error bars represent the standard error of the mean, n = 10 measurements.*

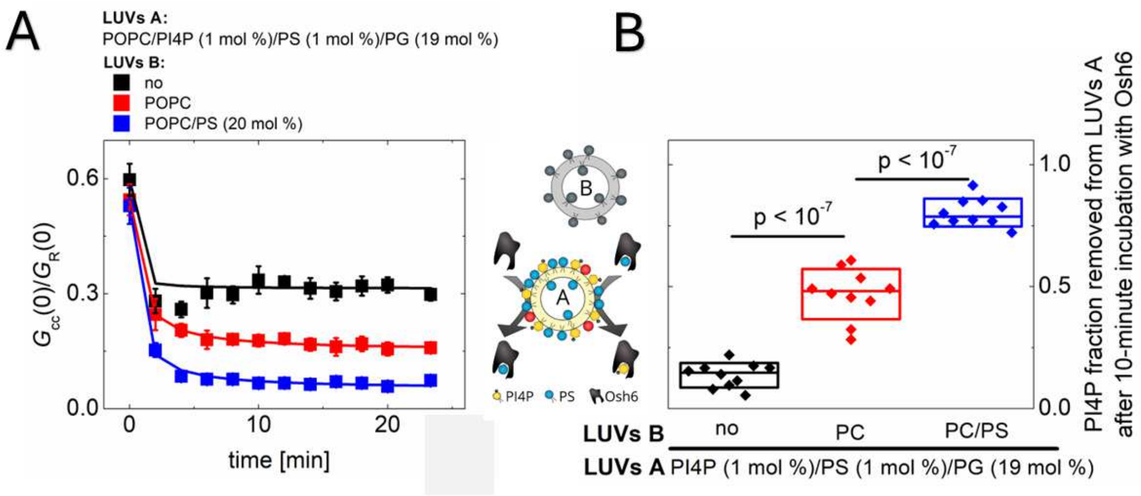

Figure S3. Synchronization of charged lipids and cargo occupancy required for the PI4P extraction from the membrane composed of POPC/PI4P (1 mol %)/PS (1 mol %)/ PS (19 mol %) – LUVs A. A) Comparison of PI4P extraction by Osh6 from LUVs A. Osh6 was preincubated with LUVs B, which i) supply Osh6 with PS, and ii) in the final mixture, serve space for PI4P release. LUVs B were composed of POPC/PS (20 mol %, blue curves), POPC (red curves) or were absent (black curves). B) Extracted fraction of PI4P within first 10 minutes of the experiment for various compositions of LUVs B given in A). *All error bars represent the standard error of the mean, n = 10 measurements*.

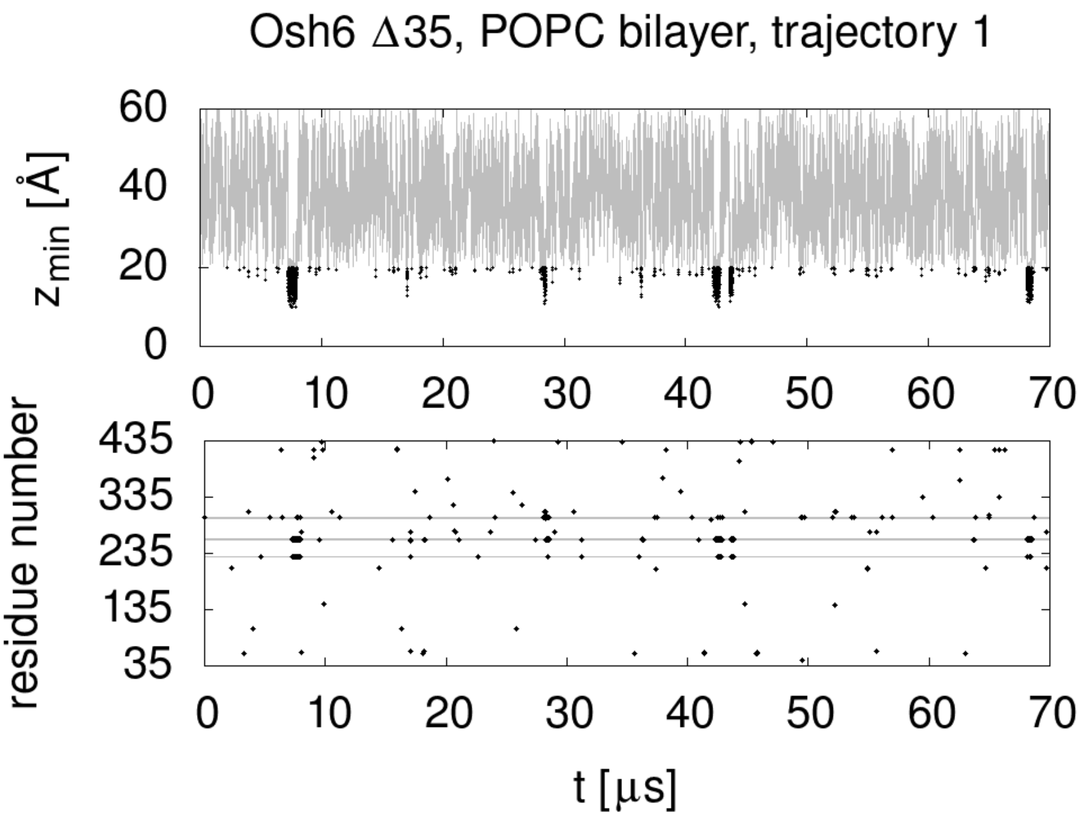

Figure S4: Results of coarse-grained MD simulations of Osh6 Δ35 with POPC membrane (trajectory 1). The upper panel shows the minimal distance z_{min} between the lipid bilayer midplane and Osh6 amino acid residues as a function of time. The points in black indicate $z_{min} < 2$ nm where at least one amino acid residue of Osh6 is in contact with the lipid bilayer. The lower panel shows which amino acid residues are in contact with the lipid bilayer during the simulation run. The horizontal lines correspond to Phe229, Tyr258, Val259, Phe260, Pro297 and Arg298.

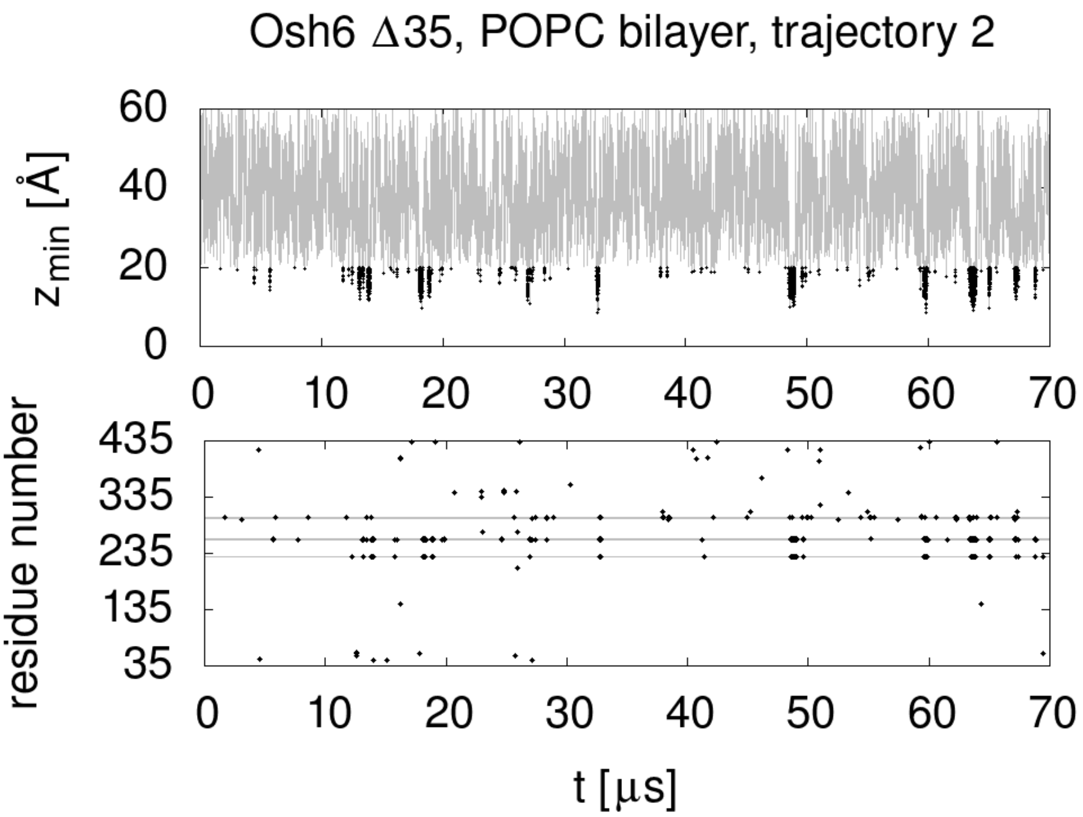

Figure S5: Analogous to figure S4 but obtained from a simulation started with different initial conditions (trajectory 2).

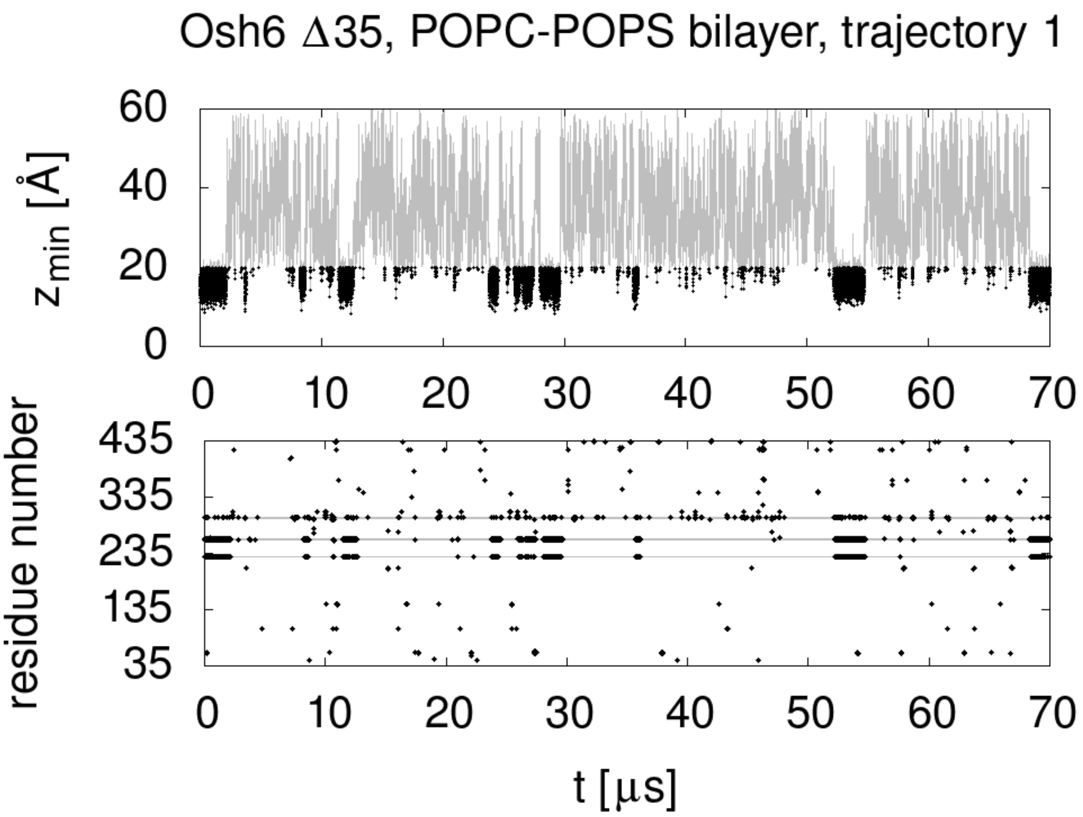

Figure S6: Results of coarse-grained MD simulations of Osh6 Δ35 with POPC-POPS membrane (trajectory 1). The upper panel shows the minimal distance z_{min} between the lipid bilayer midplane and Osh6 amino acid residues as a function of time. The points in black indicate z_{min} < 2 nm where at least one amino acid residue of Osh6 is in contact with the lipid bilayer. The lower panel shows which amino acid residues are in contact with the lipid bilayer during the simulation run. The horizontal lines correspond to Phe229, Tyr258, Val259, Phe260, Pro297 and Arg298.

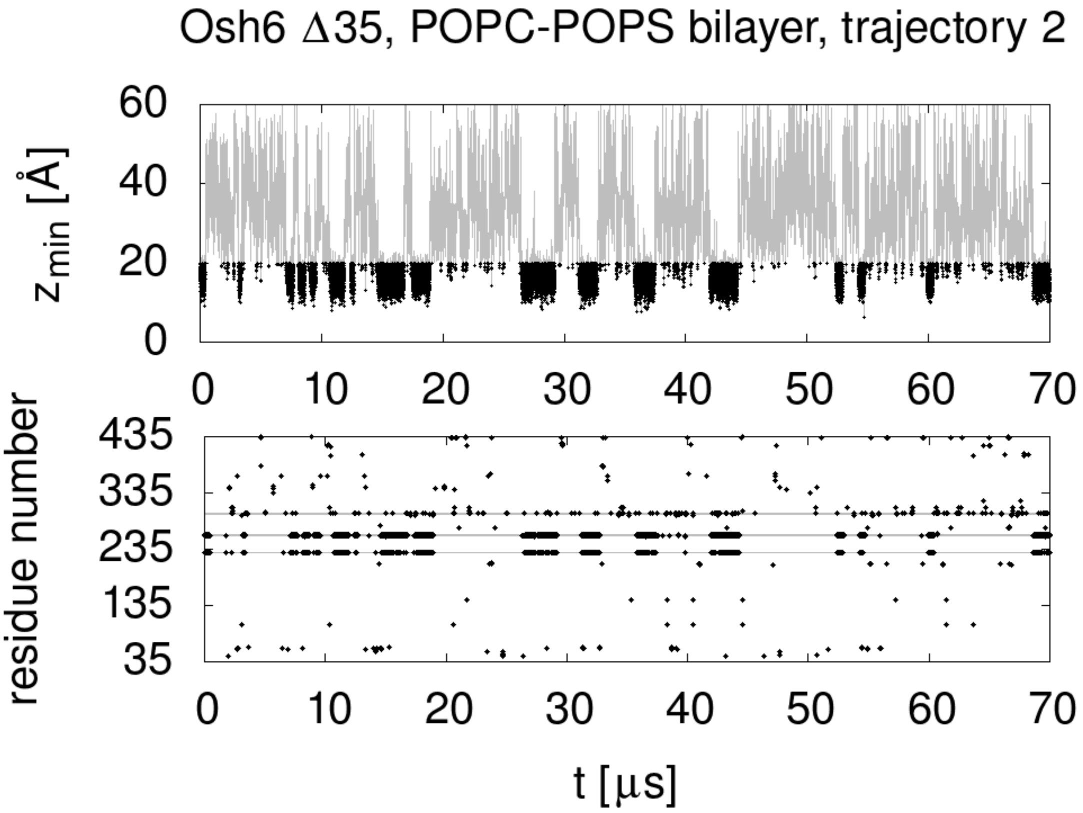

Figure S7: Analogous to figure S6 but obtained from a simulation started with different initial conditions (trajectory 2).

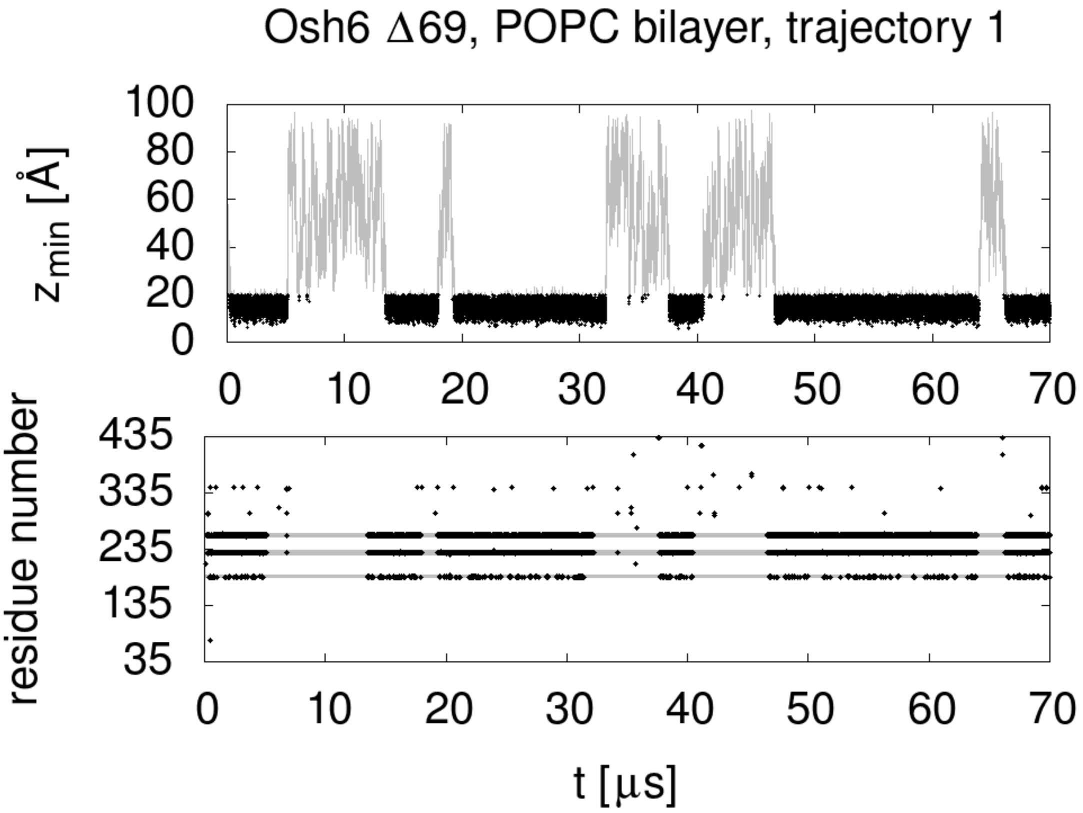

Figure S8: Results of coarse-grained MD simulations of Osh6 Δ69 with POPC membrane (trajectory 1). The upper panel shows the minimal distance z_{min} between the lipid bilayer midplane and Osh6 amino acid residues as a function of time. The points in black indicate z_{min} < 2 nm where at least one amino acid residue of Osh6 is in contact with the lipid bilayer. The lower panel shows which amino acid residues are in contact with the lipid bilayer during the simulation run. The horizontal lines correspond to regions from Arg184 to Ser189, from Arg225 to Arg233 and from Lys256 to Tyr263.

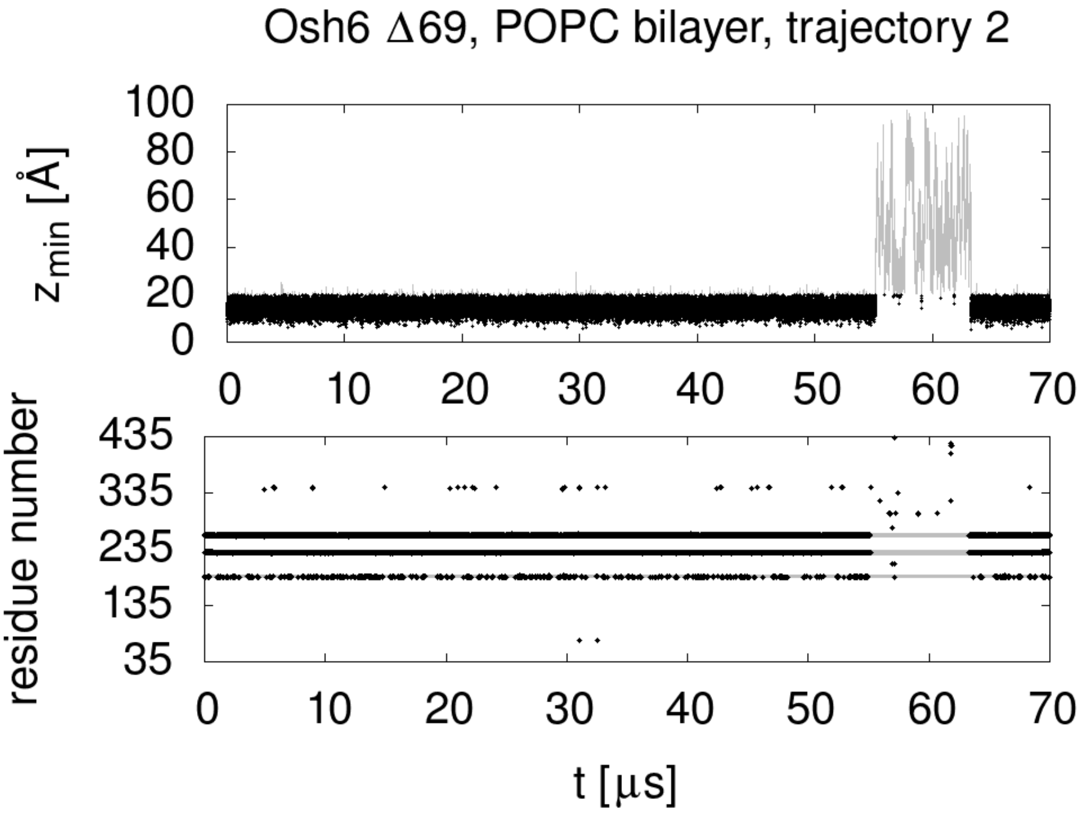

Figure S9: Analogous to figure S8 but obtained from a simulation started with different initial conditions (trajectory 2).

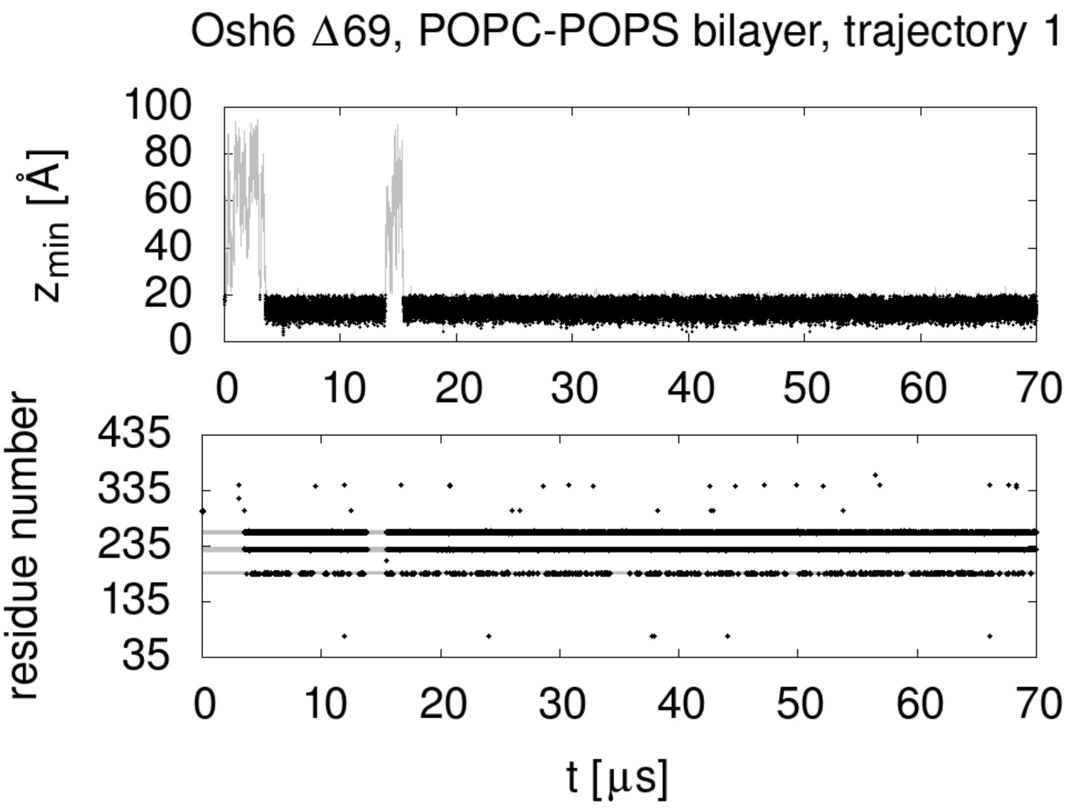

Figure S10: Results of coarse-grained MD simulations of Osh6 Δ69 with POPC-POPS membrane (trajectory 1). The upper panel shows the minimal distance z_{min} between the lipid bilayer midplane and Osh6 amino acid residues as a function of time. The points in black indicate z_{min} < 2 nm where at least one amino acid residue of Osh6 is in contact with the lipid bilayer. The lower panel shows which amino acid residues are in contact with the lipid bilayer during the simulation run. The horizontal lines correspond to regions from Arg184 to Ser189, from Arg225 to Arg233 and from Lys256 to Tyr263.

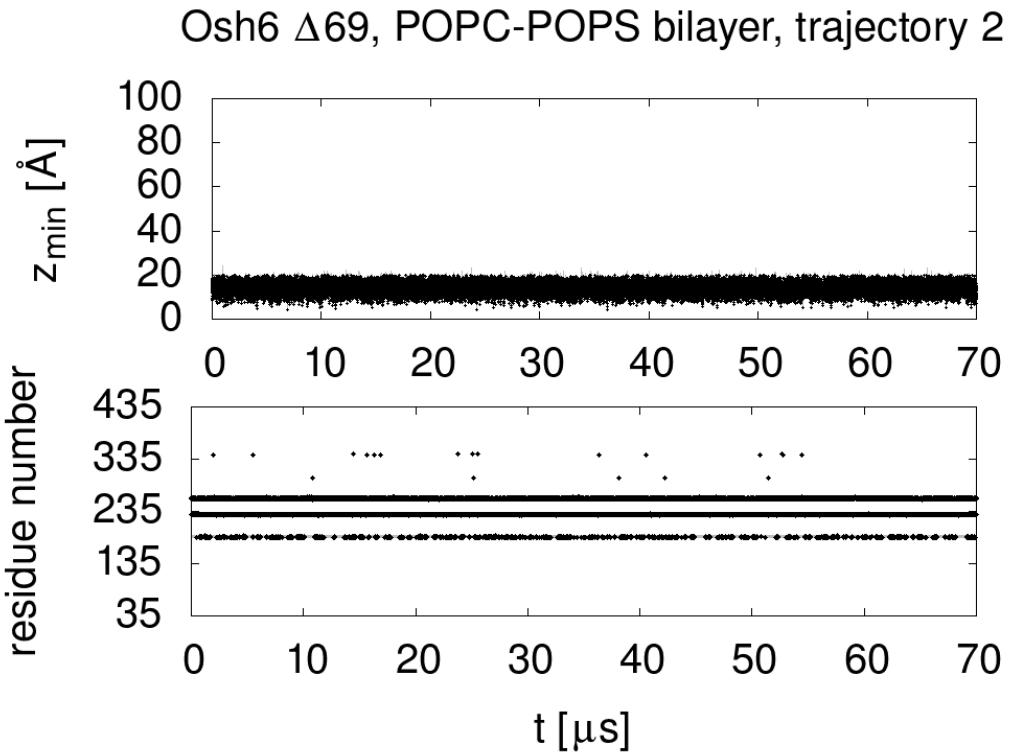

Figure S11: Analogous to figure S10 but obtained from a simulation started with different initial conditions (trajectory 2).
