# Supplementary material for: Coordination of transporter, cargo, and membrane properties during non-vesicular lipid transport
Source: Commun Biol. 2024 Nov 27;7:1585. doi: 10.1038/s42003-024-07301-3 (PMC11603022; doi:10.1038/s42003-024-07301-3)
Supplement: Supplementary file 3 — Description of Additional Supplementary File [file 42003_2024_7301_MOESM3_ESM.pdf]

Description of additional supplementary files

File name: Supplementary data

Description: Source data for Figs.2-8. Fig. 1 does not contain any experimental data.
