# Supplementary material for: Coordination of transporter, cargo, and membrane properties during non-vesicular lipid transport
Source: Commun Biol. 2024 Nov 27;7:1585. doi: 10.1038/s42003-024-07301-3 (PMC11603022; doi:10.1038/s42003-024-07301-3)
Supplement: Supplementary file 5 — nr-reporting-summary [file 42003_2024_7301_MOESM5_ESM.pdf]

# nature portfolio

Corresponding author(s): Jana Humpolickova, Alena Ballekova

Last updated by author(s): Sep 17, 2024

# Reporting Summary

Nature Portfolio wishes to improve the reproducibility of the work that we publish. This form provides structure for consistency and transparency in reporting. For further information on Nature Portfolio policies, see our Editorial Policies and the Editorial Policy Checklist.

## Statistics

For all statistical analyses, confirm that the following items are present in the figure legend, table legend, main text, or Methods section.

| n/a | Confirmed | |
|---|---|---|
| ☐ | ☒ | The exact sample size (*n*) for each experimental group/condition, given as a discrete number and unit of measurement |
| ☐ | ☒ | A statement on whether measurements were taken from distinct samples or whether the same sample was measured repeatedly |
| ☐ | ☒ | The statistical test(s) used AND whether they are one- or two-sided<br>*Only common tests should be described solely by name; describe more complex techniques in the Methods section.* |
| ☒ | ☐ | A description of all covariates tested |
| ☒ | ☐ | A description of any assumptions or corrections, such as tests of normality and adjustment for multiple comparisons |
| ☐ | ☒ | A full description of the statistical parameters including central tendency (e.g. means) or other basic estimates (e.g. regression coefficient) AND variation (e.g. standard deviation) or associated estimates of uncertainty (e.g. confidence intervals) |
| ☐ | ☒ | For null hypothesis testing, the test statistic (e.g. *F*, *t*, *r*) with confidence intervals, effect sizes, degrees of freedom and *P* value noted<br>*Give P values as exact values whenever suitable.* |
| ☒ | ☐ | For Bayesian analysis, information on the choice of priors and Markov chain Monte Carlo settings |
| ☒ | ☐ | For hierarchical and complex designs, identification of the appropriate level for tests and full reporting of outcomes |
| ☒ | ☐ | Estimates of effect sizes (e.g. Cohen's *d*, Pearson's *r*), indicating how they were calculated |

*Our web collection on statistics for biologists contains articles on many of the points above.*

## Software and code

Policy information about availability of computer code

| | |
|---|---|
| Data collection | LAS X, Leica |
| Data analysis | Las X, Matlab |

For manuscripts utilizing custom algorithms or software that are central to the research but not yet described in published literature, software must be made available to editors and reviewers. We strongly encourage code deposition in a community repository (e.g. GitHub). See the Nature Portfolio guidelines for submitting code & software for further information.

## Data

Policy information about availability of data

All manuscripts must include a data availability statement. This statement should provide the following information, where applicable:

- Accession codes, unique identifiers, or web links for publicly available datasets
- A description of any restrictions on data availability
- For clinical datasets or third party data, please ensure that the statement adheres to our policy

The minimum dataset is included in this article and its supplementary information files.

## Research involving human participants, their data, or biological material

Policy information about studies with human participants or human data. See also policy information about sex, gender (identity/presentation), and sexual orientation and race, ethnicity and racism.

| | |
|---|---|
| Reporting on sex and gender | na |
| Reporting on race, ethnicity, or other socially relevant groupings | na |
| Population characteristics | na |
| Recruitment | na |
| Ethics oversight | na |

Note that full information on the approval of the study protocol must also be provided in the manuscript.

# Field-specific reporting

Please select the one below that is the best fit for your research. If you are not sure, read the appropriate sections before making your selection.

☒ Life sciences ☐ Behavioural & social sciences ☐ Ecological, evolutionary & environmental sciences

For a reference copy of the document with all sections, see nature.com/documents/nr-reporting-summary-flat.pdf

# Life sciences study design

All studies must disclose on these points even when the disclosure is negative.

| | |
|---|---|
| Sample size | Each data point presented in the figures was analyzed from 10 technical replicates of the experiment, and each presented outcome was independently repeated at least three times. |
| Data exclusions | No data were exluded |
| Replication | Each experiment presented in the manuscript was independently repeated at least three times to ensure the reliability and reproducibility of our results |
| Randomization | Our study does not require any specific randomization process as the samples are inherently unbiased. They are not influenced by any selection criteria or preparation methods, ensuring the validity of our results without the need for additional randomization measures. |
| Blinding | Blinding was not necessary in our scientific study because the nature of our research ensures objectivity and eliminates potential biases regardless of participant or researcher awareness. Specifically, the outcomes we are measuring are objective and quantifiable, and they are not subject to subjective interpretation or influence by either the participants or the researchers. Therefore, the integrity of our results remains intact without the need for blinding procedures.<br>Blinding was not necessary in our scientific study because the nature of our research ensures objectivity and eliminates potential biases regardless of participant or researcher awareness. Specifically, the outcomes we are measuring are objective and quantifiable, and they are not subject to subjective interpretation or influence by either the participants or the researchers. Therefore, the integrity of our results remains intact without the need for blinding procedures. |

# Behavioural & social sciences study design

All studies must disclose on these points even when the disclosure is negative.

| | |
|---|---|
| Study description | na |
| Research sample | na |
| Sampling strategy | na |
| Data collection | na |
| Timing | na |
| Data exclusions | na |

| | |
|---|---|
| Non-participation | na |
| Randomization | na |

# Ecological, evolutionary & environmental sciences study design

All studies must disclose on these points even when the disclosure is negative.

| | |
|---|---|
| Study description | na |
| Research sample | na |
| Sampling strategy | na |
| Data collection | na |
| Timing and spatial scale | na |
| Data exclusions | na |
| Reproducibility | na |
| Randomization | na |
| Blinding | na |

Did the study involve field work? ☐ Yes ☐ No

## Field work, collection and transport

| | |
|---|---|
| Field conditions | na |
| Location | na |
| Access & import/export | na |
| Disturbance | na |

# Reporting for specific materials, systems and methods

We require information from authors about some types of materials, experimental systems and methods used in many studies. Here, indicate whether each material, system or method listed is relevant to your study. If you are not sure if a list item applies to your research, read the appropriate section before selecting a response.

## Materials & experimental systems

| n/a | Involved in the study | |
|---|---|---|
| ☒ | ☐ | Antibodies |
| ☒ | ☐ | Eukaryotic cell lines |
| ☒ | ☐ | Palaeontology and archaeology |
| ☒ | ☐ | Animals and other organisms |
| ☒ | ☐ | Clinical data |
| ☒ | ☐ | Dual use research of concern |
| ☒ | ☐ | Plants |

## Methods

| n/a | Involved in the study | |
|---|---|---|
| ☒ | ☐ | ChIP-seq |
| ☒ | ☐ | Flow cytometry |
| ☒ | ☐ | MRI-based neuroimaging |

## Antibodies

| | |
|---|---|
| Antibodies used | na |
| Validation | na |

# Eukaryotic cell lines

Policy information about cell lines and Sex and Gender in Research

| | |
|---|---|
| Cell line source(s) | na |
| Authentication | na |
| Mycoplasma contamination | na |
| Commonly misidentified lines (See ICLAC register) | na |

# Palaeontology and Archaeology

| | |
|---|---|
| Specimen provenance | na |
| Specimen deposition | na |
| Dating methods | na |

☐ Tick this box to confirm that the raw and calibrated dates are available in the paper or in Supplementary Information.

| | |
|---|---|
| Ethics oversight | na |

Note that full information on the approval of the study protocol must also be provided in the manuscript.

# Animals and other research organisms

Policy information about studies involving animals; ARRIVE guidelines recommended for reporting animal research, and Sex and Gender in Research

| | |
|---|---|
| Laboratory animals | na |
| Wild animals | na |
| Reporting on sex | na |
| Field-collected samples | na |
| Ethics oversight | na |

Note that full information on the approval of the study protocol must also be provided in the manuscript.

# Clinical data

Policy information about clinical studies

All manuscripts should comply with the ICMJE guidelines for publication of clinical research and a completed CONSORT checklist must be included with all submissions.

| | |
|---|---|
| Clinical trial registration | na |
| Study protocol | na |
| Data collection | na |
| Outcomes | na |

# Dual use research of concern

Policy information about dual use research of concern

## Hazards

Could the accidental, deliberate or reckless misuse of agents or technologies generated in the work, or the application of information presented in the manuscript, pose a threat to:

| No | Yes | |
|---|---|---|
| ☒ | ☐ | Public health |
| ☒ | ☐ | National security |
| ☒ | ☐ | Crops and/or livestock |
| ☒ | ☐ | Ecosystems |
| ☒ | ☐ | Any other significant area |

### Experiments of concern

Does the work involve any of these experiments of concern:

| No | Yes | |
|---|---|---|
| ☒ | ☐ | Demonstrate how to render a vaccine ineffective |
| ☒ | ☐ | Confer resistance to therapeutically useful antibiotics or antiviral agents |
| ☒ | ☐ | Enhance the virulence of a pathogen or render a nonpathogen virulent |
| ☒ | ☐ | Increase transmissibility of a pathogen |
| ☒ | ☐ | Alter the host range of a pathogen |
| ☒ | ☐ | Enable evasion of diagnostic/detection modalities |
| ☒ | ☐ | Enable the weaponization of a biological agent or toxin |
| ☒ | ☐ | Any other potentially harmful combination of experiments and agents |

## Plants

| | |
|---|---|
| Seed stocks | na |
| Novel plant genotypes | na |
| Authentication | na |

## ChIP-seq

### Data deposition

☐ Confirm that both raw and final processed data have been deposited in a public database such as GEO.

☐ Confirm that you have deposited or provided access to graph files (e.g. BED files) for the called peaks.

| | |
|---|---|
| Data access links<br>*May remain private before publication.* | na |
| Files in database submission | na |
| Genome browser session<br>(e.g. UCSC) | na |

### Methodology

| | |
|---|---|
| Replicates | na |
| Sequencing depth | na |
| Antibodies | na |
| Peak calling parameters | na |
| Data quality | na |
| Software | ma |

# Flow Cytometry

## Plots

Confirm that:

☐ The axis labels state the marker and fluorochrome used (e.g. CD4-FITC).

☐ The axis scales are clearly visible. Include numbers along axes only for bottom left plot of group (a 'group' is an analysis of identical markers).

☐ All plots are contour plots with outliers or pseudocolor plots.

☐ A numerical value for number of cells or percentage (with statistics) is provided.

## Methodology

| | |
|---|---|
| Sample preparation | na |
| Instrument | na |
| Software | na |
| Cell population abundance | na |
| Gating strategy | na |

☐ Tick this box to confirm that a figure exemplifying the gating strategy is provided in the Supplementary Information.

# Magnetic resonance imaging

## Experimental design

| | |
|---|---|
| Design type | na |
| Design specifications | na |
| Behavioral performance measures | na |

## Acquisition

| | |
|---|---|
| Imaging type(s) | na |
| Field strength | na |
| Sequence & imaging parameters | na |
| Area of acquisition | na |

Diffusion MRI ☐ Used ☐ Not used

## Preprocessing

| | |
|---|---|
| Preprocessing software | na |
| Normalization | na |
| Normalization template | na |
| Noise and artifact removal | na |
| Volume censoring | na |

## Statistical modeling & inference

| | |
|---|---|
| Model type and settings | na |
| Effect(s) tested | na |

Specify type of analysis: ☐ Whole brain ☐ ROI-based ☐ Both

Statistic type for inference

(See Eklund et al. 2016)

na

Correction

na

## Models & analysis

| n/a | Involved in the study |
| --- | --- |
| ☒ | ☐ Functional and/or effective connectivity |
| ☒ | ☐ Graph analysis |
| ☒ | ☐ Multivariate modeling or predictive analysis |

Functional and/or effective connectivity

na

Graph analysis

na

Multivariate modeling and predictive analysis

na
